# The Duality of Schizotypy: Is it Both Dimensional and Categorical?

**DOI:** 10.3389/fpsyt.2014.00134

**Published:** 2014-09-24

**Authors:** Oliver John Mason

**Affiliations:** ^1^Research Department of Clinical, Educational and Health Psychology, University College London, London, UK

**Keywords:** schizotypy, taxon, dimensions, bibliometrics, psychometrics

Schizotypy is the notion that schizophrenia-like features can form, in the absence of illness, a temperamental “type” or personality trait. Both typological and characterological accounts were present at the notion’s conception as, historically, both categorical (“Kraepelinian”) and dimensional [e.g., Kretchmer’s “schizothymic” temperament; (1)] accounts of psychotic illness have vied against one another with the former clearly ascendant in biological psychiatry at least. Paul Meehl’s influential development of the categorical account (2) theorized the “schizotype” as the category as the fundamental phenotypic foundation of “true” schizophrenia. Variants of this model remain central to theorizing in the North American tradition at least. The dimensional view, revitalized by Hans Eysenck, is best represented in contemporary theory by Gordon Claridge’s “quasi-dimensional” model (3).

In 1995, Adrian Raine and Todd Lencz (4) set out some of the theoretical and conceptual issues in schizotypal personality research and outlined the “categories versus dimensions” issue as “perhaps the most important” of all (p. 5). They suggested pursuing both approaches so as to see, which is most productive. There is of course a distinction here between *theory* and *methodology*. I aim to argue here based on four observations of the empirical literature that aspects of *both* theoretical accounts may be valid, and that a diversity of methods may have utility in the field.

Claridge’s dimensional account postulates *underlying* dimensionality of risk for illness with superimposed clinical discontinuities – the schizophrenic “spectrum” of illnesses. The critical difference between the two accounts of schizotypy lies in the non-clinical portion of the phenotype. In the categorical account, only a portion of phenotypic schizotypy is at genuine elevated risk, the “true” schizotype, the remainder is pseudophenotypic, superficially mimicking schizotypy but not possessing true genetic risk: Adrian Raine (5) termed the latter “pseudo-schizotypal.” In the dimensional account, by contrast, there is the possibility of “genuine” schizotypy possessing a healthy or adaptive outcome (6); a theme I reprise in my conclusions.

A few years ago (7), I conducted a bibliographic analysis of the schizotypy literature that evidenced the growing popularity of empirical research in the field (schizotyp* OR schizoid* OR psychosis prone*), and of experimental studies in particular. In addition, I divided the empirical literature into psychometric and experimental studies, and into those taking a categorical and dimensional approach (based on their statistical treatment). The major growth in the literature has been in experimental studies of which more have taken a dimensional (e.g., correlational) approach (Figure 1).

Clearly, there are advantages and disadvantages to both statistical approaches and this choice does not necessarily imply a strong theoretical preference. For example, most quantitative genetic studies examine correlations as a matter of course. Conversely, studies based on diagnostic procedures usually retain a categorical approach. Moreover, a minority of studies report both statistical treatments, often with broadly commensurate results. Treating schizotypy variables as continuous variables is perhaps sometimes preferred as statistical power in many analyses is likely to exceed dichotomized treatment. This is especially the case if the latter takes seriously the taxonomic prediction of 10–15% of a general population sample (arguably a “median split” is the worst of all possible worlds). Large samples are required if the truly taxonomic approach is to be taken in a multivariate analysis. While this suits some fields such as quantitative genetics, it is not suited to others such as brain imaging. On the other hand, a common strategy is to preselect “schizotypal” and “non-schizotypal” groups via large-scale screening using a psychometric instrument. This usefully reduces the number needed to test experimentally to achieve statistical power. However, the strategy may or may not imply testing of a categorical model – it is also, of course, a strategy of convenience for testing dimensional differences.

As a consequence of all these considerations, I would argue that while genuine differences clearly exist between the models theoretically, these are very rarely tested against one another genuinely at the empirical level. Evidence can be found (and is often rehearsed) for both categorical and dimensional positions – even from the same dataset. In some ways, this apparent duality may parallel the famous “wave–particle” duality of quantum theory that suggests that both accounts can be “true” in different ways, and thus seeks to explain a diverse range of observations. At the crudest level, one can observe that broad measurement of trait tendencies tend to produce continua, and narrow “symptom-focused” measures lead to categories. I would like to suggest some important ways that *both* may have validity and research utility (and I do not claim that this is an exhaustive list).

**Figure 1:**
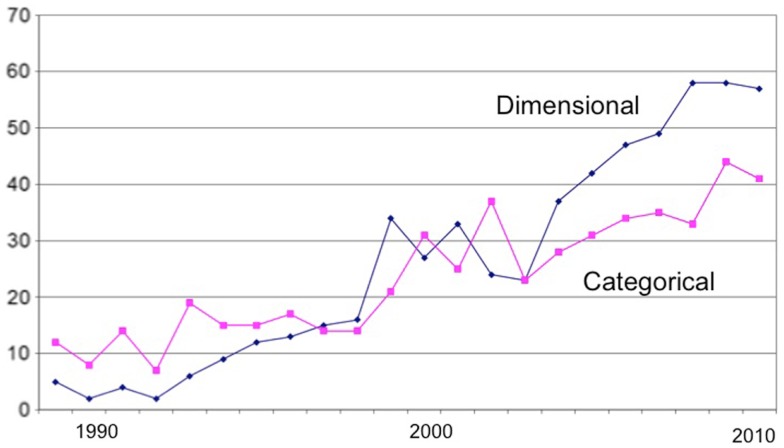
**Bibliometrics: dimensional and categorical approaches**.

I am not alone in noticing empirical evidence for both positions (8). From reviews of the epidemiological evidence of clinical disorders, Linscott and van Os suggest that there is true continuum to the non-clinical. Where I differ from their position is their suggestion that evidence in the general population suggests a latent categorical structure with “two types of people.” This structure is generally argued for as a result of attempts to identify a taxon psychometrically. However, the statistical issues of this argument certainly allow for divergent interpretations: the issue of taxonometrics in schizotypy has been much discussed with little resolution (e.g., see Personality and Individual Differences 44:8; 2008). Where I do agree with their position is in viewing schizotypy *per se* as too narrow a lens, “psychosis proneness” captures the variety of traits relevant to psychotic disorders as a whole.

## Trait Multi-Dimensionality

Regardless of psychometric arguments about putative taxons, it is likely that some measures suit one theoretical position better than another. Those with “stronger” symptom-like measures may tend to discontinuities, while others offer greater dimensionality. In addition, even the range and nature of dimensions of schizotypal personality are argued over, with perhaps the broadest consensus concerning a distinction between positive and negative schizotypy. Arguably, there are stronger indications for the taxonomic nature of negative schizotypal features such as trait anhedonia [for review see Ref. (9)]. Conversely, Edens et al. (10) found “compelling evidence in two studies of a latent dimensional structure to paranoid traits.” In general, and perhaps somewhat surprisingly, there is better evidence for the continuous distribution of “positive” schizotypy (e.g., delusional/paranoid ideation and hallucination proneness) than for “negative” schizotypy (anhedonia/social impairment).

## The Potential Utility of Schizotypal Clusters

In a development of this first point, Suhr and Spitznagel (11, 12) attempted to overcome the common inconsistency of neurocognitive findings in schizotypy by clustering schizotypal individuals rather than studying individual dimensions. Executive function deficits were selectively seen in the negative schizotypy cluster; who were also more often rated neurocognitively impaired. However, a cluster high on both positive and negative schizotypy had the most unusual social behavior ratings. Subsequently, Barrantes-Vidal et al. (13) similarly advanced evidence that clusters worked more effectively than dimensions in predicting neurocognition and neurological “soft signs.” Arguably, the confluence of dimensional traits to produce a “taxon-like” cluster may be best suited to identifying those with neurocognitive deficits, and possibly also in other experimental contexts.

## The Operation of Discontinuous “State-Like” Phenomena

While personality traits are usually seen as broadly consistent over time, stress or other unusual circumstances produce “state” effects that may possess qualitatively different, and thus discontinuous, features. In this way, traits may proceed, more or less temporarily, to “symptoms” in the absence of a diagnosed syndrome. Usually these are probably highly temporary, but where more persistent or frequent that they effectively form sub-syndromal versions of disorders such as “basic symptoms” captured by the Schizophrenia Proneness Instrument (14). These sub-syndromal symptoms may be associated with the more clearly dysfunctional cognitive, affective, and behavioral features of schizotypy/schizophrenia. As they can become quite persistent states, they may well give the appearance of a taxon.

## Epistatic Mechanisms Mediating Gene–Environment Interaction

It is increasingly accepted that many individual loci each make a very small contribution to overall genetic risk (15). On *prima facie* grounds, such evidence supports the notion of one or more continua (16, 17) and probably underpins the heritability seen for broadly defined schizotypal traits. However, there remains the possibility for individual schizotypal features to arise from more specific gene loci, or more likely from complex gene-gene and gene-environment interactions. Overall, it is difficult to disambiguate continuous from discontinuous genetic effects from studies of heritability alone. One of the largest heritability studies to date (18), albeit with no single standard psychometric scale, suggested a pattern of heritability for social anhedonia consistent with a single dominant gene as postulated in the Meehlian account. Overall, many heritability studies [e.g., Ref. (19)] postulate heritability of around 50% with the remainder due to non-shared environmental variance. While the quest for a “schizophrenia gene” able to discriminate clinical from non-clinical groups continues with linkage and genome-wide association studies, there has been little sustained success: Weinberger concluded that results “are decidedly disappointing to those expecting this strategy to yield conclusive evidence of common variants predicting risk for schizophrenia” [p. 840, Ref. (20)]. A small number of gene-of-interest (GOI) studies have nevertheless some consistent results largely with positive schizotypy. These concern the polymorphisms of genes relevant to dopamine transmission such as COMT (16, 21), DRD1 and DRD2 (22), SLC6A3 (16, 21), or MAOA (16). Such studies evidence greater schizotypy associating with several polymorphisms such as rs4680 SNP (single nucleotide polymorphisms) within the COMT-gene in a continuous fashion. However, sometimes this association is only seen in the presence of an environmental factor such as childhood abuse (23). As investigation of these in detail is in its infancy, it is likely that much greater specification of their relevance and mode of action will occur in future studies.

There is also increasing evidence of epigenetic action, whereby environmental factors influence the expression of genes (15, 24). Svarkic et al. [p. 2, Ref. (25)] outline a model, whereby “abnormal epigenetic states with large effects are superimposed on a polygenic liability to schizophrenia.” This is effectively an extension or variant of point 3 and highlights how the actions of specific genes (individually making a small quantitative contribution to risk) may translate into genuinely taxonomic discontinuities – *but only in the context of a pathogenic environment*.

## Conclusion

Overall, I have attempted to argue that *even in the non-pathological* domain of schizotypal individual differences there are numerous possibilities for both dimensional and categorical expressions both of traits and states. Taxonomic expression has greater support for negative schizotypal features such as anhedonia and potentially some associated neurocognitive features; positive schizotypy, on the other hand, sees much empirical support for “true” dimensionality at both genotype and phenotypic expression. Even here, however, there is room for gene–environment interactions and epigenetics to produce discontinuous results.

As a rider to this final point, it is apposite to point out that there may equally be important phenotypic consequences for schizotypy in the absence of a pathogenic environment or the presence of a protective factor such as high cognitive or emotional intelligence. Thus, positive schizotypy is also associated with a range of “healthy” or at least adaptive outcomes. Again, paralleling the advantages seen with cluster analytic approaches, Tabak and Weisman de Mamani (26) identified several schizotypal latent profiles: the negative/disorganized schizotypy profile had the poorest levels of well-being and schizotypes solely with positive features had the highest – commensurate with non-schizotypes. Taking a similar latent profile analytic approach to a non-clinical sample, Hori et al. (27) described 15% as “high-positive-schizotypy/adaptive” and possessing of high self-directedness, cooperativeness, and self-transcendence. This is consistent with growing evidence of the highly creative and spiritual outcomes for some schizotypal individuals (28, 29), and may point to the operation of antagonistic pleiotropy or genetic linkage such that schizotypal traits survived throughout our evolutionary history (30).

## Conflict of Interest Statement

The author declares that the research was conducted in the absence of any commercial or financial relationships that could be construed as a potential conflict of interest.
